# Occupational Conditions and Well‐Being Among Informal Waste Pickers in Hong Kong: A Comparative Study of 2018 and 2023

**DOI:** 10.1002/ajim.70134

**Published:** 2026-09-12

**Authors:** Siu‐Ming Chan, Alan Tam, Heng Xu, Yu‐tian He, Yuen‐Ki Tang, Gum‐Ryeong Park, Wing‐Him Tang

**Affiliations:** ^1^ Department of Social and Behavioural Sciences City University of Hong Kong Hong Kong SAR China; ^2^ Department of Health Policy and Management Korea University Seoul Republic of Korea; ^3^ Waste Picker Platform, New Arrivals Limited Hong Kong SAR China

**Keywords:** Hong Kong, informal waste pickers, occupational condition, physical health

## Abstract

**Background:**

Informal waste pickers play a crucial role in urban recycling systems but often face precarious employment and systematic marginalization. This study examines the evolving demographic, occupational, and health trends of informal waste pickers in Hong Kong between 2018 and 2023.

**Methods:**

This study utilized a face‐to‐face structured questionnaire to collect cross‐sectional survey data in 2018 (*N* = 505) and 2023 (*N* = 701) across Hong Kong. Independent‐samples *t*‐tests were conducted to compare demographic variables, working conditions, income sources and levels, physical health, and negative encounters (e.g., property theft and scale manipulation) between the two periods.

**Results:**

The surveys show that the waste picker population remains predominantly older adults. Between 2018 and 2023, their living conditions exhibited significant changes across four core dimensions. First, the average monthly income from recycling and the proportion relying on the Old Age Living Allowance increased significantly. Second, a greater number of waste pickers reported being motivated by earning a basic livelihood and supporting environmental protection. Third, the proportion experiencing weighing‐scale manipulation and physical injury increased. Finally, despite a significant increase in daily work rounds, the proportion reporting physical strain declined.

**Conclusions:**

Despite nominal income gains, Hong Kong's predominantly female waste pickers face intensified structural violence via unfair markets and discrimination. Hence, female‐friendly policies and other procedures, including sector formalization, the establishment of waste picker organizations, intermediary regulation, retirement and health support, and anti‐discrimination public education, are suggested.

## 1 Introduction

### 1.1 Background of Informal Waste Pickers in Hong Kong

Population aging has become a major social and public health concern, especially in contexts where later‐life employment is insecure, and retirement protection is limited. In Hong Kong, despite its high‐income economy, not all older adults are equally protected from financial hardship in later life, and some continue to rely on low‐paid or informal work to meet basic needs [1, 2]. One example of this work is informal waste picking, referring to the collection of recyclable materials from waste by workers outside formal systems. Although their work contributes to urban waste recovery, it is usually carried out without stable earnings, labor protection, or occupational safeguards [3, 4], thereby exposing workers to economic insecurity and health risks. In Hong Kong, many informal waste pickers are older adults, especially women. For many of them, waste picking often serves as a way to supplement inadequate income in later life because pensions and old‐age social welfare may be insufficient to meet basic living needs, rather than providing a stable or socially recognized occupation in its own right [2, 5]. The persistence of this form of work in a highly urbanized city, where large volumes of waste are generated and formal waste management systems coexist with informal practices, highlights broader structural inequalities related to an aging population, poverty, and insecure work [2].

This activity constitutes an integral component of the infrastructure through which recyclable materials are identified, collected, sorted, transported, and reintegrated into commodity markets, rather than a marginal or residual practice. International research indicates that millions of individuals depend on informal waste recovery; their labor reduces the volume of waste requiring municipal collection and disposal, and supports resource recovery and the circular economy [3, 6]. Informal waste pickers participate in the everyday governance of waste recovery by making practical decisions about material value, negotiating access to materials and space with residents, businesses, recycling intermediaries, and enforcement agencies, and absorbing costs and risks otherwise externalized by formal systems. These contributions are environmental, economic, and political, even if unrecognized in official accounts of urban waste management.

Existing research shows that this group faces multiple disadvantages. Waste picking as a part of sanitation work is physically demanding, and workers often involuntarily do it in an insecure and stigmatized environment [7, 8]. A qualitative study describes bodily strain, declining physical capacity, family tension, and exclusion in everyday work settings [2]. Also, quantitative research further shows that difficult work experiences, including physical strain, theft, rude treatment, and government enforcement actions, are associated with higher levels of depression and anxiety symptoms [5]. There is also evidence of internal inequality within the group, especially by gender, socioeconomic position, income, working conditions, and psychological health [1]. In the past five years, the Hong Kong Government has implemented a series of policies to safeguard income through engaging in waste recycling activities. Relevant local NGOs have designed and implemented interventions to improve the physical and psychosocial well‐being of older waste pickers, while also promoting public education to challenge stigma and build a more picker‐friendly community [9]. At the same time, the COVID‐19 pandemic and subsequent lockdown policies posed significant challenges to the survival and routine work of informal waste pickers. During the pandemic period, informal waste pickers were not just more exposed to the risk of being infected; they were more likely to be misperceived and dispelled by the government and the general public as hygiene and health risk factors, which in turn led to exclusion [2].

It is notable that the period between our two surveys spans the COVID‐19 pandemic and its aftermath, providing an opportunity to analyze the effects of a major urban disruption. The pandemic influenced consumption and packaging habits, access to public spaces, labor market conditions, public health regulations, and perceptions of the legitimacy of street‐based livelihoods [10, 11, 12]. It also revealed the complex status of waste pickers: their role in material recovery remained environmentally essential, yet their presence was at times regulated as a potential nuisance or public health concern [12]. For this analysis, the pandemic is treated as a contextual rupture, rather than a causal intervention suitable for quasi‐experimental estimation, to explore how relationships among waste pickers, recycling shops, communities, and public authorities may have changed between 2018 and 2023.

### 1.2 Research Gap

Although existing literature has documented the health, social, and economic difficulties faced by informal waste pickers, three gaps remain. First, informal waste picking is often examined primarily as an individual livelihood or health issue, leaving its systemic contribution to urban recycling and its position within relations of governance, exchange, and enforcement underdeveloped [3, 6, 13]. Second, most Hong Kong studies rely on cross‐sectional data and offer a snapshot at a single time point [1, 5, 9]. Third, the pandemic has rarely been used as an analytical lens for examining how urban disruptions reshape waste streams, labor‐market dependence, public‐space control, intermediary power, and collective capacity among informal waste pickers [12].

To address this gap, this study compares survey data collected in 2018 and 2023 to examine changes in the working, living, and health‐related conditions of informal waste pickers in Hong Kong in the context of policy interventions and pandemic‐related disruptions. Specifically, this study asks whether there have been significant changes in their demographic and household characteristics, working conditions and income sources, adverse occupational experiences, and self‐reported physical and mental health between the two periods.

As the surveys are based on distinct cross‐sectional samples rather than longitudinal tracking of the same individuals, the analysis focuses on population‐level differences between the two survey periods. It does not measure individual change, nor does it assign causal attribution for observed differences to COVID‐19 or to any specific policy intervention.

## 2 Methods

### 2.1 Data and Sample

The investigation drew upon data from the 2023 Hong Kong Waste Pickers Research Report, a joint endeavor among multiple academic entities and nongovernmental organizations. Oversight of this collaborative effort was provided by the leading author and principal investigator of the research team. The City University of Hong Kong's Human Subjects Ethics Sub‐Committee granted ethical approval for this research (Ref: HU‐STA‐00000994). Informed consent was obtained in street‐based settings, where the study details were thoroughly explained to participants and their comprehension confirmed before their voluntary consent was secured. Data collection was executed meticulously through structured questionnaires, delivered face‐to‐face at 84 recycling shops and 11 recycling trolleys across all 18 districts. Data gathering took place daily from 9:00 a.m. to 4:00 p.m., spanning from November 18 to November 24, 2023. Operational management and survey execution were conducted by 320 volunteer instructors, who were organized into teams of two to three interviewers per site, to enhance the efficiency of data collection. The survey phase culminated with 914 respondents engaged. Inclusion criteria included both male and female waste pickers active in street or building settings, whereas the exclusion criterion was set for individuals who stored collected waste at home.

Initially, respondents were categorized based on their recycling practices into two groups: those recycling at home and those involved in recycling within community streets or buildings. This analysis focused on the latter, targeting individuals active in community recycling settings. From the initial pool of respondents, 100 individuals solely involved in home recycling were excluded. Moreover, 103 individuals opted out of the survey, and 10 were excluded due to providing inadequate responses, which was defined as answering fewer than five questions. Following these exclusions, the analysis was conducted with a sample of 701 valid questionnaires.

### 2.2 Measurement

In both the 2018 and 2023 surveys examining the characteristics and well‐being of those termed “cardboard grannies” in Hong Kong, measurements included demographic variables, the conditions of waste‐picking work, the physical health of the waste pickers, and various incidents encountered during their waste‐picking activities.

#### 2.2.1 Demographic Variables

Both surveys collected basic demographic information such as age, gender, marital status, and educational background. Additionally, respondents were queried about their living status through four specific questions: “Are you living alone?”, “Are you living with your spouse?”, “Are you living with your children?”, and “Are you living with other family members?” Responses to these inquiries were recorded as either “yes” or “no.”

#### 2.2.2 Conditions of Waste‐Picking Work

Specific parameters measured included the number of years engaged in waste‐picking, the number of days worked per week, the number of hours worked per day, and the number of rounds made daily to collect items, capturing key dimensions of occupational characteristics, including work experience and work intensity. Additional data we captured included the reasons for engaging in waste‐picking, the types of materials collected, and the monthly income derived from these activities.

#### 2.2.3 Incidents Encountered During Waste‐Picking

The survey included a multi‐select question that allowed respondents to identify if they had experienced any of the following situations: being chased away or fined by the Food and Environmental Hygiene Department, confiscation of their belongings, accidents occurring during waste‐picking hours, theft or robbery of collected items, rude treatment from citizens or shop owners, and being cheated by recycling shops in the weight of collected materials.

#### 2.2.4 Physical and Mental Health

Health outcomes were categorized into two domains: physical and mental health. The survey incorporated specific variables to assess the physical condition of the waste pickers. The first question inquired whether the respondents experienced any strain or illness directly from their long‐term engagement in informal waste picking. The second question addressed the presence of any chronic diseases among the participants. For both inquiries, respondents were allowed to answer with either “yes” or “no.” Mental health status was assessed using the Patient Health Questionnaire‐2 (PHQ‐2) and the Generalized Anxiety Disorder 2‐item scale (GAD‐2). Both instruments are brief, validated screening tools measuring core symptoms of depression and anxiety over the past 2 weeks. Each scale consists of two items rated on a 4‐point Likert scale (0 = *not at all* to 3 = *nearly every day*), with total scores ranging from 0 to 6. Higher scores indicate greater depressive or anxiety symptom severity.

### 2.3 Statistical Analysis

The statistical analysis of the data collected from the 2018 and 2023 surveys was performed using SPSS version 29.0. For continuous variables, such as the number of hours worked per day or monthly income from waste‐picking, the analysis used independent‐samples *t‐*tests. For categorical variables, which include responses to questions about physical health conditions (yes/no), incidents encountered (multiple selections possible), and reasons for engaging in waste‐picking (multiple selections possible), chi‐square tests of independence were used.

## 3 Results

### 3.1 Demographic and Household Characteristics

Table S1 compares the demographic and household characteristics of informal waste pickers in 2018 and 2023. Female respondents remained predominant in both years, encompassing 80% and 81.4% of the sample, respectively. Moreover, the age distribution differed significantly between 2018 and 2023 (*χ* ^2^ = 30.57, *p* < 0.001). In both years, older adults constituted the majority of respondents. The largest age group in both 2018 and 2023 was those 70–79 years old, which accounted for 38.8% and 37.9% of respondents, respectively.

Regarding living arrangements, the proportion of respondents living with a partner increased significantly from 26.9% in 2018 to 36.7% in 2023 (*χ* ^2^ = 12.553, *p* < 0.001), whereas the proportion living with relatives decreased from 21.6%to 12.9% (*χ* ^2^ = 15.589, *p* < 0.001). No significant difference was observed in the proportions of respondents living alone and living with children between 2018 and 2023. Significant changes were observed in residential building type (*χ* ^2^ = 76.64, *p* < 0.001). For housing types, the percentage of respondents living in self‐owned public housing increased from 6.8% in 2018 to 14.6% in 2023, and home ownership rose from 2.0% to 6.8%. Meanwhile, the proportion of respondents renting public housing was 41.5% in 2023, down from 46.3% in 2018, and the proportion of homeless respondents dropped from 6.8% in 2018 to 0.7% in 2023.

### 3.2 Types of Recyclables Collected and Adverse Work Experiences

Analysis of the data from Table S2 for the years 2018 and 2023 reveals several statistically significant changes in the types of recyclables collected and adverse work experiences. In assessing encounters with foam glue, a notable increase in the percentage of affirmative responses was observed from 6.2% in 2018 to 13.1% in 2023 (*χ* ^2^ = 15.261, *p* < 0.001). In the category of waste plastic/glue bottles, the percentage of affirmative responses significantly rose from 6% in 2018 to 22.7% in 2023 (*χ* ^2^ = 62.065, *p* < 0.001). With regard to adverse experiences, physical injuries caused by sorting or moving recyclables saw a significant increase in affirmative responses from 27.4% in 2018 to 37% in 2023 (*χ* ^2^ = 11.417, *p* < 0.001). The responses related to weighing‐scale manipulation by recyclers showed a substantial shift, with affirmative answers significantly increasing from 26.8% in 2018 to 51.7% in 2023 (*χ* ^2^ = 66.617, *p* < 0.001). In contrast, the proportion reporting that recyclables or personal belongings had been stolen rose slightly from 9.3% to 11.0%, although this difference was not statistically significant (*χ* ^2^ = 0.897, *p* = 0.344). Similarly, the proportion reporting rude treatment by citizens increased from 9.9% to 13.6%, but such a change remained nonsignificant (*χ* ^2^ = 3.634, *p* = 0.057).

### 3.3 Income Sources and Monthly Income

Table S3 compares income sources and monthly income of informal waste pickers in 2018 and 2023. A significant change was observed in the receipt of Old Age Living Allowance (OALA) (*χ* ^2^ = 23.845, *p* < 0.001). In 2018, 84.5% of respondents did not receive this allowance, whereas in 2023, this figure decreased significantly to 72.1%, indicating a higher uptake of the allowance. Conversely, the percentage of respondents who received the allowance significantly increased from 15.5% in 2018 to 27.9% in 2023. For income from work other than informal waste picking, there was also a notable change (*χ* ^2^ = 23.495, *p* < 0.001). The proportion of respondents who did not have other sources of income increased from 67.8% in 2018 to 80.7% in 2023. Correspondingly, the percentage of people who had other sources of income significantly decreased from 32.2% in 2018 to 19.3% in 2023. Moreover, the distribution of total monthly income also changed significantly over time (*χ* ^2^ = 100.88, *p* < 0.001). The lowest income bracket of HK$0–500 declined slightly from 5.7% in 2018 to 4.9% in 2023. There is a notable increase in the higher income brackets. For instance, the proportion of individuals earning between HK$4001–5000 rose from 8.8% in 2018 to 17.3% in 2023. Similarly, the proportion of those earning $10,001 or more increased from 9% to 20.5% in the same period. For monthly recycling income, the mean income in 2018 was HK$716.4, which significantly increased to HK$1566.3 in 2023. The *t*‐test statistic of 5.153 (*p* value < 0.001) revealed a significant increase in the mean monthly income.

### 3.4 Working Time, Work Intensity, and Output in Informal Waste Picking

Table S4 provides a comparative analysis of time and effort in informal waste picking between the years 2018 and 2023. For the average daily work rounds, there was an increase from 1.9 in 2018 to 2.64 in 2023, a statistically significant increase. For average recycling weight collected each time, measured in kilograms, there was a significant decrease from 35.06 kg in 2018 to 30.14 in 2023 (*t* = −2.745, *p* = 0.006).

### 3.5 Reasons for Engaging in Waste Picking

Table S5 shows significant changes in the reasons individuals engage in informal waste picking between 2018 and 2023. For the category of earning a basic livelihood, the percentage of respondents who did not engage in informal waste picking for this reason decreased from 75.5% to 69.1%, while those who engaged in informal waste picking to earn a livelihood significantly increased from 24.5% to 30.9% (*χ* ^2^ = 6.054, *p* = 0.014). In the context of maintaining physical activity to prevent body degeneration, the percentage of respondents who did not consider this a reason for engaging in informal waste picking decreased markedly from 74% to 60%. Correspondingly, the percentage who engage in informal waste picking for this purpose increased from 26% to 40% (*χ* ^2^ = 25.505, *p* < 0.001). Finally, the data regarding support for environmental protection showed a decrease from 74.2% to 58.3% in respondents who did not choose to be informal waste pickers for this reason. Meanwhile, the percentage of respondents who chose to be informal waste pickers to support environmental protection rose from 25.8% to 41.7% (*χ* ^2^ = 32.587, *p* < 0.001).

### 3.6 Self‐Reported Physical Health Conditions

Table S6 presents changes in self‐reported physical conditions between 2018 and 2023. Several indicators of physical strain and pain showed significant declines over time. The prevalence of sensitive skin decreased from 11% in 2018 to 4.9% in 2023 (*χ* ^2^ = 12.383, *p* < 0.001). Low back pain also showed a significant decrease (*χ* ^2^ = 19.588, *p* < 0.001), with reported cases dropping from 52.9% in 2018 to 38.8% in 2023. Similarly, foot pain prevalence significantly decreased (*χ* ^2^ = 65.271, *p* < 0.001) from 63.9% in 2018 to 36.3% in 2023. Hand pain also saw a significant decrease (*χ* ^2^ = 17.178, *p* < 0.001), falling from 48.1% of respondents reporting it in 2018 to 34.2% in 2023. Furthermore, a statistically significant decrease was observed in the reported prevalence of neck pain (*χ* ^2^ = 9.423, *p* = 0.002), from 20.6% in 2018 to 13% in 2023. Finally, the prevalence of shoulder pain showed a statistically significant change (*χ* ^2^ = 8.05, *p* = 0.005), with 31% reporting it in 2018 compared to 22.5% in 2023.

## 4 Discussion

This study aimed to examine changes in the characteristics, working conditions, and health of informal waste pickers between 2018 and 2023. The key findings of this study show that, compared with 2018, informal waste picking in 2023 is increasingly driven by the need to earn a basic livelihood, maintain physical activity to slow physical decline, and contribute to environmental protection, with these motivations rising significantly. The proportion of collected materials—particularly foam glue and plastic/glue bottles—has also increased significantly. Informal waste pickers report more adverse experiences, especially injuries during sorting or transport and disputes over weighing by recyclers, both of which increased significantly. Reports of theft of recyclables or personal belongings and rude treatment by citizens also increased, although these changes were not statistically significant.

Taken together, these changes suggest a reorganization rather than an unequivocal improvement in informal waste picking. An increase in collection rounds, reduced average weight per trip, greater recovery of packaging materials, and higher recycling income signal ongoing shifts in waste streams and work patterns. Concurrently, the notable rise in reported incidents of scale manipulation and workplace injuries suggests that economic gains have been accompanied by heightened vulnerability to intermediary influence and occupational hazards. Waste pickers remain in a precarious position within the recycling chain: while they supply materials to downstream traders, they often lack access to transparent pricing, reliable weighing systems, secure storage and sorting facilities, and effective collective bargaining structures. Accordingly, the analysis situates individual well‐being within the broader organization of the urban recycling market, rather than attributing vulnerability solely to the characteristics of individual workers. This interpretation aligns with local qualitative research indicating that prices and payments for recyclables are mediated by depot‐specific practices, such as weight measurement and quality evaluation [2]. These operational dynamics offer a plausible context for understanding how increased recycling income may occur alongside more frequent work rounds and a rise in reported scale manipulation.

Significant differences are observed in demographic characteristics, particularly age, living with partner, and type of residence. Although long‐term engagement in informal waste picking remains associated with physical strain (e.g., skin sensitivity and pain in the shoulders, lower back, neck, feet, and hands), the proportion reporting these health problems declined significantly in 2023. These findings underscore the profession's core values of dignity and empowerment: even under structural constraints, older waste pickers demonstrate remarkable resilience and adaptive capacity. However, this observed decline should be interpreted with caution, as there remains a possibility of underreporting of pain, particularly among older waste pickers who may normalize or downplay physical discomfort.

We also found that income patterns have shifted, with increases in OALA and total monthly income, but a decline in income from work other than informal waste picking. In addition, informal waste pickers in 2023 made more daily collection trips and collected less weight per trip in pounds.

The research reveals that females and older age groups remain the majority of informal waste pickers over the 5‐year period, which might signal underlying structural issues that contribute to this phenomenon. This could be linked to women's willingness to prioritize their roles as caregivers, placing children and family ahead of their own personal career development [14]. Their contribution to the family may exacerbate their disadvantage as workers due to exclusion from development [15], pushing them further into the cycle of poverty [16, 17]. Furthermore, the concentration of younger women in lower‐skilled jobs during the 1970s and 1980s [18, 19] may have limited their ability to adapt to Hong Kong's subsequent economic transformations, particularly the decline of the industrial sector [20], leaving some at a continued disadvantage in the labor market [21]. Further investigation is expected to reinforce the above conjecture, particularly regarding education attainment and potential life patterns among female informal waste pickers.

Interestingly, while the number of informal waste pickers driven by earning a basic livelihood increased, the proportion of those motivated by preventing body degeneration and environmental protection increased even more. This may suggest a gradual shift in the perceived social value of informal waste picking, potentially contributing to its future formalization. However, more efforts are needed to eliminate the general public's bias against informal waste picking, as the percentage of informal waste pickers treated rudely by citizens rose slightly from 9.9% to 13.6%. As Landsberger argues, the portrayal of waste pickers as “down and out” obscures their agency and social contribution [22]. Deconstructing such deficit‐based narratives is therefore essential to reframing informal waste pickers as active environmental actors whose labor sustains urban recycling systems. A qualitative investigation conducted among older waste pickers in Hong Kong elucidates the interplay of multiple motivations: participants characterized waste picking as a source of modest income, an avenue for physical activity, and a means of sustaining personal purpose [2]. Consequently, the observed survey changes may be better interpreted as reflecting the intertwined livelihood and psychosocial significance of waste picking, rather than a simple progression toward formalization.

Monthly recycling income was higher in the 2023 sample than in the 2018 sample, increasing from HK$716 to HK$1566. This temporal pattern should not be interpreted as a causal effect of the pandemic or as evidence that poverty was alleviated [23, 24, 25]. The increase may reflect a combination of more intensive collection, changes in recyclable volumes and composition, variation in market prices, and reduced access to other employment. Approximately 18% of respondents in 2023 reported 1–3 years of waste‐picking experience, a period overlapping with the pandemic; this is consistent with, but does not prove, pandemic‐related entry into the activity. Research indicates that pandemic unemployment was concentrated among economically disadvantaged older workers [26], while increased use of takeaway packaging and personal protective equipment changed urban waste streams [10, 11, 12, 27]. The statistically significant increases in the collection of foam packaging and plastic bottles are compatible with this contextual explanation, although the surveys cannot isolate the contribution of each mechanism.

Although the proportion of respondents having OALA as their source of income increased, it is noteworthy that less than 30% of the 80% of respondents who are eligible for OALA do take advantage of it. This implies OALA has not effectively benefited the elderly in need, rendering its resources ineffective. It is noteworthy that 29.2% and 47.6% of respondents reported having no expectation for support from the government and NGOs, respectively, and over 70% stated that they never participated in group activities. This lack of expectation of social activities may reflect a self‐perception of social isolation among informal waste pickers, given the inherently solitary nature of their work.

For health outcomes, while average daily working rounds have increased significantly from 2018 to 2023, the substantial decline in the proportion of informal waste pickers experiencing physical strain and pain presents an intriguing paradox. Additionally, mean scores on the PHQ‐2 and GAD‐2 are 0.86 and 0.83, respectively, indicating a relatively low risk of depression and anxiety among respondents. The combination of findings implies that informal waste pickers' physical and mental health may be improving despite increased workloads. In contrast, several studies suggest that a potential relationship may exist between informal waste picking and common mental disorders [28, 29]. Local qualitative research further reveals that older waste pickers often perceive walking and sustained physical activity as advantageous, even as they endure the physical strain associated with bending, lifting, sorting, and transporting recyclables [2]. This concurrent experience of both perceived benefits and occupational burdens may help account for the observed inconsistency, though the present repeated cross‐sectional data are insufficient to directly evaluate this mechanism.

Local qualitative research demonstrates that waste picking is intimately connected to neighborhood social dynamics and patterns of urban spatial access. Older waste pickers described receiving assistance from local businesses and residents, yet simultaneously encountered precarious conditions for sorting and storing materials, social stigma, competition with fellow pickers, and the risk of confiscation by enforcement officials [2]. These findings imply that the negative experiences identified in the survey arise not solely from individual vulnerabilities, but are also shaped by everyday interactions with community members, recycling intermediaries, and urban authorities.

## 5 Policy Implication

Effective policy should start with the recognition that informal waste pickers contribute a public environmental service within Hong Kong's waste management system. Instead of requiring individual workers to fit a predefined model of formal employment, policy ought to address the institutional arrangements that currently shift market, health, and regulatory risks onto these workers. As such, formalization should be approached as a negotiated and plural process, potentially encompassing legal recognition, service contracts, collective organization, social protection measures, and guaranteed access to materials and workspace [3, 6, 13, 30, 31, 32].

International experience cautions that formalization is not inherently inclusive. Although Colombia is frequently regarded as a pioneer in inclusive recycling, formalization requirements in Bogotá and Cartagena have produced material, spatial, and organizational forms of exclusion for workers unable to meet administrative standards [33]. Related research from São Paulo shows that co‐production arrangements may privilege larger, formally‐recognized cooperatives while excluding street‐based waste pickers [34]. Formalization can also reduce waste pickers to low‐paid service providers while overlooking their practical knowledge of material identification and segregation [35]. These findings suggest that formalization in Hong Kong should be evaluated by whether it protects livelihoods, autonomy, access to recyclables, and participation in decision‐making, rather than by registration or contractual status alone.

First, waste pickers should be acknowledged as urban environmental workers and relevant policy stakeholders. Public agencies ought to involve representatives of this group in processes related to recycling facilities, municipal solid waste policies, street management, and urban redevelopment. Recognition should extend to their substantive contributions to material recovery and their detailed understanding of local waste flows [36, 37, 38]. The incorporation of formal recycling spaces, rest areas, access to water and sanitation, and designated storage or trolley‐parking zones into neighborhood planning is especially important in proximity to wet markets and other locations with high recyclable output [39, 40, 41].

Second, regulation of market intermediaries is essential to promote distributive fairness. The increase in reported scale manipulation, which ranges from 26.8% to 51.7%, highlights the necessity of interventions directed at exchange structures, rather than placing responsibility solely on workers. Recycling shops should employ certified, prominently displayed scales, post current purchase prices, issue clear transaction records, and participate in accessible complaint and inspection mechanisms. Supplementary measures, such as shared weighing stations or mobile verification services, could further address information asymmetry. These steps would help to reduce the bargaining imbalance between individual waste pickers and recycling intermediaries.

Third, collective organization should be supported, but not required, as a prerequisite for assistance. Government and nongovernmental organizations ought to allocate resources to waste‐picker‐led associations, mutual‐aid groups, or cooperatives that represent workers, share information on prices and rights, negotiate spatial access, organize collective purchasing of equipment, and facilitate connections to legal, social, and health services [30, 31, 42]. Comparative research shows that waste picker organizations can generate grassroots innovations, provide environmental services, strengthen economic stability, and build political influence through local, national, and transnational networks [43, 44]. Cooperative experience also demonstrates potential to improve waste pickers' position in recycling value chains, although unequal access to state contracts and organizational resources may favor larger entities over independent workers [34, 45]. Support must therefore protect organizational autonomy and remain accessible to unorganized workers, who may face barriers related to mobility, time, language, or trust. Where waste pickers are incorporated into circular‐economy or extended producer responsibility initiatives, participatory governance and recognition of their social, environmental, and knowledge contributions should be explicit design principles [35, 46, 47]. Collective organization functions not only as a means of individual empowerment but more fundamentally as a mechanism for altering structures of voice and power within the recycling sector.

Fourth, universal and occupation‐specific social and health protections should be established. Considering the workforce's age and gender composition, there is a clear need for improved retirement income, streamlined access to the OALA, preventive health care, physiotherapy, injury referral services, and affordable protective equipment. These supports should not be contingent on ceasing waste picking. For workers seeking alternative employment, voluntary training and caregiver‐friendly opportunities may broaden available options; however, labor‐market activation by itself cannot resolve issues such as low recyclable prices, unstable public spaces, or inequitable market dynamics.

Fifth, governance approaches should move away from punitive or stigma‐based practices in favor of clear, proportionate regulations. Guidelines ought to differentiate legitimate public safety risks from the routine spatial needs associated with collecting, sorting, and temporarily storing recyclables. Enforcement personnel should be adequately trained, confiscation protocols should incorporate notification and retrieval safeguards, and there should be independent avenues for complaint review. Public education initiatives should be developed collaboratively with waste pickers, emphasizing their expertise as environmental actors rather than depicting them as passive beneficiaries of charity [48].

These recommendations represent a move from traditional, individual‐focused formalization toward broader structural inclusion. The central premise is that advances in well‐being depend on redistributing recognition, infrastructure, information, protections, and decision‐making authority throughout the urban recycling system.

### 5.1 Limitations

The data collection for this study was conducted within a defined and relatively short period. Although this approach allowed for efficient and systematic data gathering, it may not fully capture potential seasonal variations in waste generation, fluctuations in recyclable market prices, or changes in work patterns that could occur across different times of the year. Future research employing longitudinal or multi‐season data collection could further enrich understanding of these dynamics. Also, the sampling approach may limit the representativeness of the findings, as participants were recruited from selected recycling sites and may not fully capture more hidden or less accessible waste pickers. Relatedly, the survey was administered by a large team of trained volunteer instructors working in small groups. While this arrangement enhanced coverage across districts and improved accessibility to participants, minor variations in questionnaire administration may have occurred despite standardized training procedures. Such variations are common in large‐scale field surveys and are unlikely to substantially affect the overall patterns observed, but they should nevertheless be acknowledged. Overall, while these methodological considerations suggest areas for further refinement in future studies, they do not detract from the robustness of the findings derived from a large and geographically comprehensive sample of informal waste pickers in Hong Kong.

The two surveys employ repeated cross‐sectional designs involving different participants. Variations in sampling locations, respondent characteristics, methods of questionnaire administration, prevailing market prices, or other unmeasured contextual factors could account for observed changes. Thus, the study design does not qualify as a quasi‐experiment and cannot attribute differences between 2018 and 2023 to COVID‐19, policy actions, or any single variable. Instead, the pandemic serves as an interpretive framework and a periodizing reference point. Furthermore, the survey did not gather data on organizational membership, collective initiatives, direct interactions with public authorities, price‐setting mechanisms, or nuanced experiences of spatial regulation. Investigating these processes requires longitudinal, qualitative, and participatory research in collaboration with waste pickers and their representative organizations.

## Author Contributions

Siu‐Ming Chan, as the first author, was responsible for conceptualization, methodology, research design and data collection, writing the original draft, reviewing and editing, supervision, and project administration. Alan Tam was involved in reviewing and editing. Heng Xu was involved in writing the original draft, reviewing and editing, and performing formal analysis. Yu‐tian He participated in writing the original draft, reviewing and editing, and performing formal analysis. Yuen‐Ki Tang participated in performing formal analysis, investigation, and reviewing and editing. Gum‐Ryeong Park focused on reviewing and editing. Wing‐Him Tang participated in research design and data collection.

## Funding

The authors have nothing to report.

## Disclosure

The authors have nothing to report.

## Ethics Statement

This study was approved by the Human Subjects Ethics Sub‐Committee of City University of Hong Kong (Ref: HU‐STA‐00000994) on May 9, 2023.

## Consent

Informed consent was obtained from each participant in the survey.

## Conflicts of Interest

The authors declare no conflicts of interest.

## Supporting information


Supporting File

## Data Availability

The data that support the findings of this study are available from the corresponding author upon reasonable request.
