# Supplementary material for: Occupational Conditions and Well‐Being Among Informal Waste Pickers in Hong Kong: A Comparative Study of 2018 and 2023
Source: Am J Ind Med. 2026 Sep 12;69(12):1037–45. doi: 10.1002/ajim.70134 (PMC13740939; doi:10.1002/ajim.70134)
Supplement: Supplementary file 1 [file AJIM-69-1037-s001.docx]

| **Appendices**  **Table I.** Comparative results of demographic and household characteristics between 2018 and 2023. | | | | | | |
| --- | --- | --- | --- | --- | --- | --- |
|  | 2018 | | 2023 | | χ² | *p* |
|  | N | % | N | % |  |  |
| **Gender** |  |  |  |  |  |  |
| Male | 101 | 20 | 126 | 18.6 | 0.345 | 0.557 |
| Female | 404 | 80 | 550 | 81.4 |  |  |
| **Age** |  |  |  |  |  |  |
| 30-39 | 2 | 0.4 | 2 | 0.3 | 30.57*** | < .001 |
| 40-49 | 5 | 1 | 6 | 0.9 |  |  |
| 50-59 | 10 | 2 | 11 | 1.6 |  |  |
| 60-69 | 74 | 14.7 | 80 | 11.8 |  |  |
| 70-79 | 195 | 38.8 | 256 | 37.9 |  |  |
| 80-89 | 128 | 25.5 | 249 | 36.8 |  |  |
| 90 or above | 72 | 14.3 | 63 | 9.3 |  |  |
| **Living Condition** | |  |  |  |  |  |
| **Living alone** |  |  |  |  |  |  |
| No | 377 | 74.7 | 475 | 70.6 | 2.392 | 0.122 |
| Yes | 128 | 25.3 | 198 | 29.4 |  |  |
| **Living with partner** |  |  |  |  |  |  |
| No | 369 | 73.1 | 426 | 63.3 | 12.553*** | < .001 |
| Yes | 136 | 26.9 | 247 | 36.7 |  |  |
| **Living with children** |  |  |  |  |  |  |
| No | 331 | 65.5 | 406 | 60.3 | 3.353 | 0.067 |
| Yes | 174 | 34.5 | 267 | 39.7 |  |  |
| **Living with relatives** |  |  |  |  |  |  |
| No | 396 | 78.4 | 586 | 87.1 | 15.589*** | < .001 |
| Yes | 109 | 21.6 | 87 | 12.9 |  |  |
| **Residential building** |  |  |  |  |  |  |
| self-owned public housing | 34 | 6.80 | 98 | 14.6 | 76.64*** | < .001 |
| Home Ownership Home Ownership | 10 | 2.00 | 46 | 6.8 |  |  |
| Own private building | 100 | 20.10 | 121 | 18.0 |  |  |
| Rent public housing | 230 | 46.30 | 279 | 41.5 |  |  |
| Rent a private building (whole unit) | 42 | 8.50 | 41 | 6.1 |  |  |
| Rent a private building (subdivided room/board room/bed) | 27 | 5.40 | 44 | 6.5 |  |  |
| Homeless | 34 | 6.80 | 5 | 0.7 |  |  |

*Note.* N = frequency.

* p < .05. ** p < .01. *** p < .001.

| **Table II.** Comparative results of types of recyclables collected and adverse work experiences between 2018 and 2023. | | | | | | | | | | |
| --- | --- | --- | --- | --- | --- | --- | --- | --- | --- | --- |
|  | | 2018 | | 2023 | | | | χ² | | *p* |
|  | | N | % | N | | | % |  |  |  |
| **Foam glue** | |  |  |  | | |  |  | |  |
| No | | 472 | 93.8 | 605 | | | 86.9 | 15.261*** | | < .001 |
| Yes | | 31 | 6.2 | 91 | | | 13.1 |  |  |  |
| **Waste plastic/glue bottles** |  | | | |  |  | |  |  | |
| No | | 474 | 94 | 538 | | | 77.3 | 62.065*** | | < .001 |
| Yes | | 30 | 6 | 158 | | | 22.7 |  |  |  |
| **Recycling or personal property stolen** |  | | | |  |  | |  |  | |
| No | | 431 | 90.7 | 608 | | | 89 | 0.897 | | 0.344 |
| Yes | | 44 | 9.3 | 75 | | | 11 |  |  |  |
| **Physical injuries caused by sorting or moving recyclables** |  | | | |  |  | |  |  | |
| No | | 344 | 72.6 | 423 | | | 63 | 11.417*** | | < .001 |
| Yes | | 130 | 27.4 | 248 | | | 37 |  |  |  |
| **Being treated rudely by citizens** |  | | | |  |  | |  |  | |
| No | | 428 | 90.1 | 577 | | | 86.4 | 3.634 | | 0.057 |
| Yes | | 47 | 9.9 | 91 | | | 13.6 |  |  |  |
| **Scale manipulated by recycler** |  | | | |  |  | |  |  | |
| No | | 346 | 73.2 | 276 | | | 48.3 | 66.617*** | | < .001 |
| Yes | | 127 | 26.8 | 296 | | | 51.7 |  |  |  |

*Note.* N = frequency.

* p < .05. ** p < .01. *** p < .001.

| **Table III.** Comparative results of income sources and monthly income between 2018 and 2023. | | | | | | |
| --- | --- | --- | --- | --- | --- | --- |
|  | 2018 | | 2023 | | χ² | *p* |
|  | N | % | N | % |  |  |
| **Old Age Living Allowance** |  |  |  |  |  |  |
| No | 410 | 84.5 | 426 | 72.1 | 23.845*** | < .001 |
| Yes | 75 | 15.5 | 165 | 27.9 |  |  |
| **Income from work other than scavenging** |  |  |  |  |  |  |
| No | 329 | 67.8 | 477 | 80.7 | 23.495*** | < .001 |
| Yes | 156 | 32.2 | 114 | 19.3 |  |  |
| **Monthly income** |  |  |  |  |  |  |
| HK$0-500 | 28 | 5.7 | 33 | 4.9 | 100.88*** | < .001 |
| HK$501-1,000 | 22 | 5.4 | 40 | 6 |  |  |
| HK$1,001-2,000 | 58 | 11.9 | 64 | 9.6 |  |  |
| HK$2,001-3,000 | 76 | 15.5 | 53 | 7.9 |  |  |
| HK$3,001-4,000 | 84 | 17.2 | 45 | 6.7 |  |  |
| HK$4,001-5,000 | 43 | 8.8 | 116 | 17.3 |  |  |
| HK$5,001-10,000 | 134 | 27.4 | 160 | 23.9 |  |  |
| HK$10,001 or More | 44 | 9 | 137 | 20.5 |  |  |
|  | M | SD | M | SD | *t* | *p* |
| **Monthly recycling income (HK$)** | 716.44 | 1052.08 | 1566.3 | 2290.1 | 5.153 | < .001 |

*Note.* N = frequency; M= mean; SD = standard deviation.

* p < .05. ** p < .01. *** p < .001.

| **Table IV.** Comparative results of working time, work intensity, and output between 2018 and 2023. | | | | | | |
| --- | --- | --- | --- | --- | --- | --- |
|  | 2018 | | 2023 | | *t* | *p* |
|  | Mean | SD | Mean | SD |  |  |
| Average daily work rounds | 1.9 | 1.2 | 2.64 | 2.5 | 6.312 | < .001 |
| Recycling weight each time (kg) | 35.06 | 30.16 | 30.14 | 27.16 | -2.745 | 0.006 |

*Note.* SD = standard deviation.

| **Table V.** Comparative results of reasons for engaging in waste picking between 2018 and 2023. | | | | | | |
| --- | --- | --- | --- | --- | --- | --- |
|  | 2018 | | 2023 | | χ² | *p* |
|  | N | % | N | % |  |  |
| **Earn basic livelihood** |  |  |  |  |  |  |
| No | 380 | 75.5 | 480 | 69.1 | 6.054* | 0.014 |
| Yes | 123 | 24.5 | 215 | 30.9 |  |  |
| **Let the body be depressed so that the**  **body will not degenerate so quickly** |  |  |  |  |  |  |
| No | 373 | 74 | 417 | 60 | 25.505*** | < .001 |
| Yes | 131 | 26 | 278 | 40 |  |  |
| **Support environmental protection** |  |  |  |  |  |  |
| No | 374 | 74.2 | 405 | 58.3 | 32.587*** | < .001 |
| Yes | 130 | 25.8 | 290 | 41.7 |  |  |

*Note.* N = frequency.

* p < .05. ** p < .01. *** p < .001.

| **Table VI.** Comparative results of physical health conditions between 2018 and 2023. | | | | | | |
| --- | --- | --- | --- | --- | --- | --- |
|  | 2018 | | 2023 | | χ² | *p* |
|  | N | % | N | % |  |  |
| **Sensitive skin** |  |  |  |  |  |  |
| No | 276 | 89 | 642 | 95.1 | 12.383*** | < .001 |
| Yes | 34 | 11 | 33 | 4.9 |  |  |
| **Shoulder pain** |  |  |  |  |  |  |
| No | 214 | 69 | 523 | 77.5 | 8.05** | 0.005 |
| Yes | 96 | 31 | 152 | 22.5 |  |  |
| **Low back pain** |  |  |  |  |  |  |
| No | 143 | 46.1 | 413 | 61.2 | 19.588*** | < .001 |
| Yes | 167 | 52.9 | 262 | 38.8 |  |  |
| **Neck pain** |  |  |  |  |  |  |
| No | 246 | 79.4 | 587 | 87 | 9.423** | 0.002 |
| Yes | 64 | 20.6 | 88 | 13 |  |  |
| **Foot pain** |  |  |  |  |  |  |
| No | 112 | 36.1 | 430 | 63.7 | 65.271*** | < .001 |
| Yes | 198 | 63.9 | 245 | 36.3 |  |  |
| **Hand pain** |  |  |  |  |  |  |
| No | 161 | 51.9 | 444 | 65.8 | 17.178*** | < .001 |
| Yes | 149 | 48.1 | 231 | 34.2 |  |  |

*Note.* N = frequency.

* p < .05. ** p < .01. *** p < .001.
